# Eligibility of Athletes With a Difference in Sex Development in Elite Sport: Opinions of National, Elite and World Class Athletes

**DOI:** 10.1002/ejsc.12300

**Published:** 2025-04-28

**Authors:** Niall Timothy Fife, Alex Lauren Shaw, Georgina Kate Stebbings, Marie Chollier, Luke Thomas Joseph Cox, Andy Neil Harvey, Alun Gwen Williams, Shane Micheal Heffernan

**Affiliations:** ^1^ Faculty of Science and Engineering Applied Sports Science Technology and Medicine Research Centre (A‐STEM) Swansea University Swansea UK; ^2^ Manchester Metropolitan Institute of Sport Manchester Metropolitan University Manchester UK; ^3^ Social and Political Science University of Chester Chester UK; ^4^ Institute of Sport, Exercise and Health University College London London UK

**Keywords:** competition, ethics, policy, sociology

## Abstract

There have been limited studies allowing key stakeholders the opportunity to voice their opinions on DSD athlete participation in elite sport. The purpose of this study was to survey athletes eligible to compete in the female category regarding DSD athletes' inclusion. This study surveyed national, elite and world class athletes (*n* = 147) competing in the female category regarding their opinions on the eligibility of DSD athletes in elite sport. The study compared current and retired Olympic sport athletes, elite versus world class athletes and current Olympic sport versus current Olympic‐recognised sport athletes. Most athletes believed that it was an unethical requirement to medicate in order to comply with eligibility regulations (67.2%). Overall, athletes did not support a separate category for DSD athletes, an opinion most strongly held for precision sports (69.5%) and a majority believed their participation in the female category was fair (54.4%, precision sports). This opinion was more commonly held by Olympic‐recognised sport than current Olympic sport athletes, particularly for sports heavily reliant on physical capacity (61.1% vs. 20%). More current Olympic sport athletes believed that the eligibility of DSD athletes for the female category was unfair, compared to Olympic‐recognised sport athletes. Athletes agreed that sports federations could be doing more to make sport more inclusive for DSD athletes (82.2%), with only 8.2% believing such athletes were treated fairly. After reviewing these novel results, the athletes' voice (in combination with scientific evidence) should be utilised to create appropriate policies that align with the collective values of athletes.

**Summary**

- This study is the first of its kind and shows that the majority of high‐level athletes surveyed believed the current treatment of DSD athletes who wish to compete in the female category was unfair, athletes medicating to* fit eligibility criteria was unethical and sporting federations need to be more inclusive.
- Opinions differed depending on sporting context, the type and level of sport and whether the athlete was retired or currently competing.
- This study provides perspectives from key stakeholders that will aid sport federations when creating/amending policies relating to the eligibility of DSD athletes in elite sports.

## 1 Introduction

Since the 1900s, separate sex categories have ensured a fair competition across sport (Elsas et al. 2000). Owing to media reports and concerns from athletes of ‘hyper masculine’ competitors, in 1966 the International Olympic Committee (IOC) introduced medical examinations to determine the sex of individuals and decide their eligibility to compete in the female category (Elsas et al. 2000). Mandatory sex verification was suspended by the IOC for the 2000 Olympic Games due to pressures from policymakers, medical professionals and women's sports advocates, who voiced concerns associated with inaccuracies, stigmatisation and the recognition that athletes' privacy was threatened through such testing (Elsas et al. 2000). In response, sport federations turned to suspicion‐based medical examination (Brömdal et al. 2020). One such high profile case was in 2009, Caster Semenya whose 800 m world championship performances raised suspicion, and her suggested masculine appearance provoked further enquiry of her sex. This resulted in Semenya being banned from competing due to her difference in sex development (DSD) (Buzuvis 2010). These are a spectrum of complex congenital conditions, characterised by atypical development of chromosomal, gonadal and/or anatomical sex (Lee et al. 2016). Each specific DSD has a myriad of causal allelic variants, resulting in varied atypical sex phenotypes (Batista and Mendonca 2022; Loch Batista et al. 2020; Zidoune et al. 2022). Of particular interest to elite sports are athletes with 46, XY karyotype, including partial and complete androgen insensitivity (PAIS/CAIS) and 5‐alpha‐reductase type 2 deficiency (5AR2D), where many cases individuals are recorded as female at birth (Loch Batista et al. 2020; Bowman‐Smart et al. 2024; World Athletics 2023). Following challenges, World Athletics agreed that Semenya had to lower her testosterone concentration below 10 nmol/L for six months before competing, via medication usually only used for health purposes (WMA 2019). In June 2010, Semenya was able to compete again after medically lowering her testosterone. However, in 2011, hyperandrogenism was introduced into the World Athletics female category regulations (International Association of Athletics Federation 2011) but was suspended by the Court of Arbitration for Sport (CAS) due to a challenge by Indian sprinter Dutee Chand in 2014 (Court of Arbitration for Sport 2014). As a result of this ruling, World Athletics introduced a further new regulation that only affected 46, XY DSD individuals with testosterone levels above 5 nmol/L (World Athletics 2018). This regulation was only enacted if the athlete wished to compete in specific running events, from 400 m to one mile (World Athletics 2018). Following appeals, this regulation was upheld, but the panel agreed that the science was not conclusive and encouraged World Athletics to take more time to do research before implementing the regulations.

In 2021, the IOC released a framework of 10 principles on gender identity and sex variations (International Olympic Committee 2021), some of which have been well received, such as Principle 6 ‘evidence‐based approach’ and Principle 8 ‘stakeholder‐centred approach’. However, some principles have recently been contested (Lundberg et al. 2024). After the IOC framework was released, sports federations, including World Athletics and World Aquatics, changed their eligibility policies concerning DSD athletes (World Athletics 2023; Aquatics 2023). However, despite calls from the IOC for more evidence and a stakeholder‐centred approach, there is currently no peer‐reviewed evidence collating key stakeholder opinions and beliefs regarding the eligibility of athletes with DSDs in the elite female category.

Policymakers have a moral obligation to develop policies that strive to find a balance between all stakeholders affected by a sport federation's actions, including the opinions of athletes currently competing in the female category (Mazanov 2016). It is essential to be aware of these potential differences in beliefes as many federation policy committees include retired athletes, and they have only recently begun to consider the ‘athlete voice’ (International Olympic Committee 2023). In addition, Olympic sport athletes gain more spectatorship, media coverage and financial benefits than athletes from Olympic‐recognised sports (Кropyvnytska et al. 2021; Litchfield 2018; Smart 2018), and competitors at the highest athletic level (> 0.00006% of all athletes (McKay et al. 2022)) have the most significant potential to gain or lose financial rewards and sponsorships (Smart 2018). Therefore, their opinions may be incongruent with those devoid of this status, and it is crucial to investigate if differences exist between groups where these benefits are abundant and those that have less access to rewards, for example Olympic‐recognised sports (International Olympic Committee 2024a), as has been shown in an alternative context recently (Shaw et al. 2024).

The primary aim of this study was to survey the opinions of national, elite and world class athletes eligible to compete in the female category regarding the eligibility of DSD athletes in elite sport. The second aim was to investigate potential differences in these opinions between Olympic and Olympic‐recognised sports, retired and current athletes and athletes competing at different levels.

## 2 Materials and Methods

### 2.1 Procedure and Questionnaire

As part of the differences in sex development and transgender elite sports (DATES) study (Shaw et al. 2024), an invitation email was distributed to Olympic‐recognised International sport federations via personal networks and social media platforms (International Olympic Committee 2024a; International Olympic Committee 2024b). The email included a link to the study's online anonymous survey (LimeSurvey Version 2.64.3 + 170,327). Purposive snowballing sampling was also used as elite athletes are a ‘hard to reach’ population (Valerio et al. 2016). Data were collected from August 2021 to August 2022. The Faculty of Science and Engineering Research Ethics and Governance Committee, Swansea University, granted ethical approval (SU‐Ethics‐Staff‐210622/486).

**FIGURE 1:**
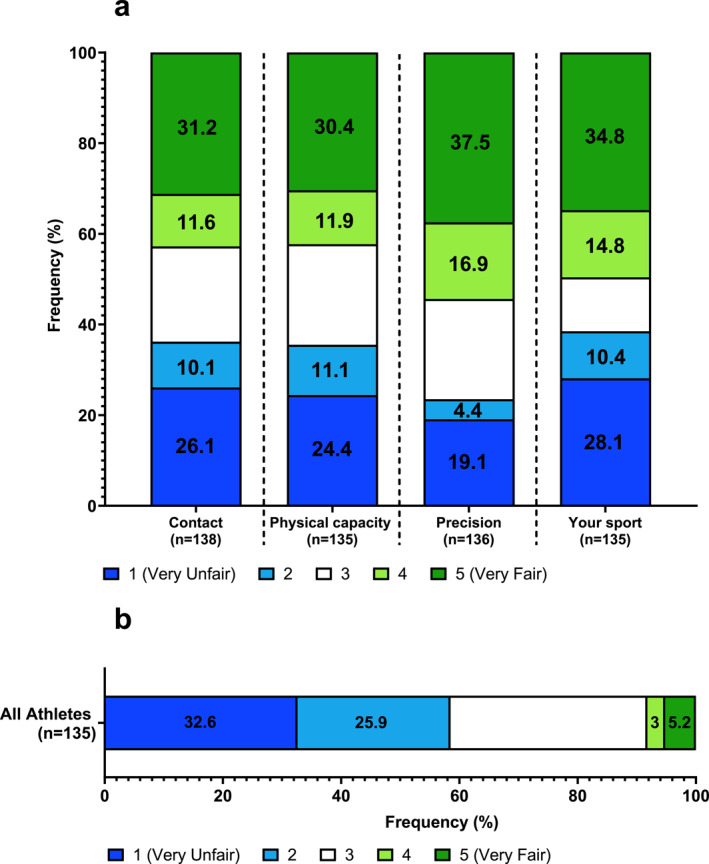
Responses of all athletes. (a) How fair is it for an athlete with a DSD to compete in the elite female category (contact/HRPC/precision/your sport). (b) How fairly do you think athletes with a DSD get treated across all sports? Some bars are ± 0.1% due to the rounding of mean.

**FIGURE 2:**
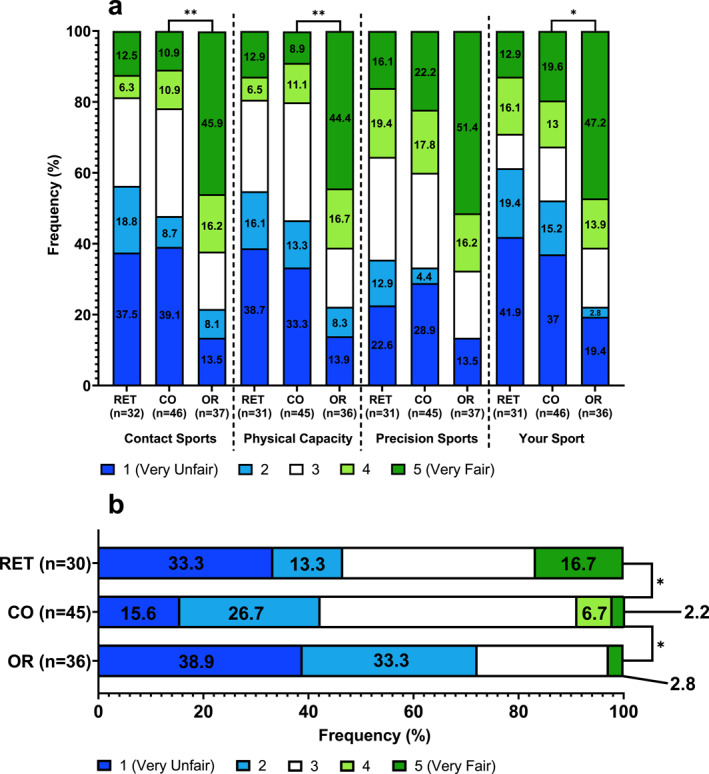
Responses of retired Olympic sport (RET), current Olympic sport (CO) and current Olympic‐recognised sport (OR) athletes. (a) How fair is it for an athlete with a DSD to compete in the elite female category in contact/HRPC/precision/your sport? (b) How fairly do you think athletes with a DSD get treated across all sports? Statistical differences are indicated by **p < 0.01 and *p < 0.05. Some bars are ± 0.1% due to the rounding of mean.

**FIGURE 3:**
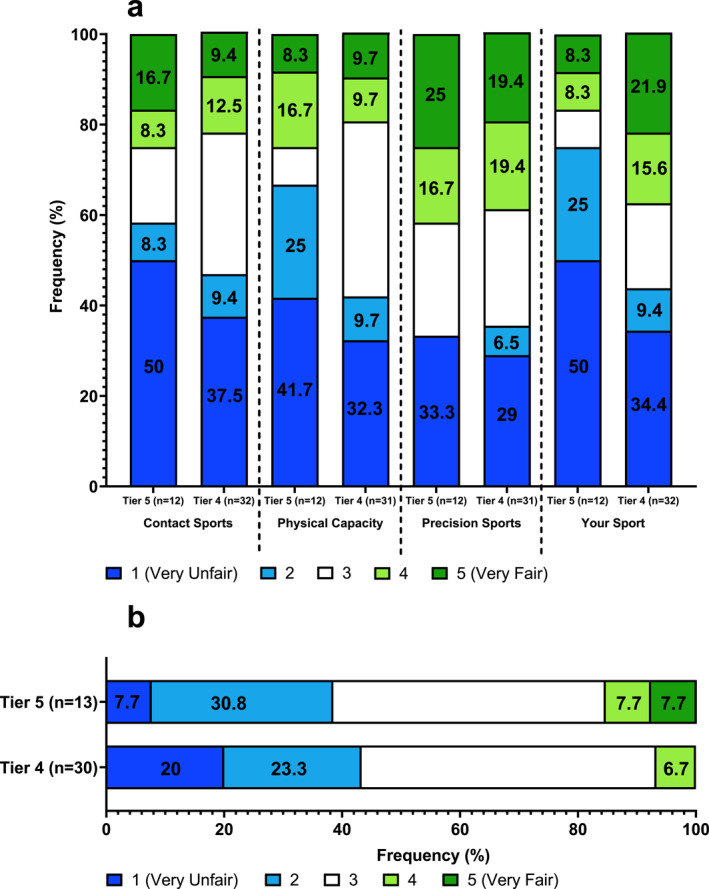
Responses of current Olympic sport (CO) athletes competing at world class (Tier 5) and elite (Tier 4) competitive standards. (a) How fair is it for an athlete with a DSD to compete in the elite female category in contact/HRPC/precision/your sport? (b) How fairly do you think athletes with a DSD get treated across all sports? Some bars are ± 0.1% due to the rounding of mean.

Participants were presented with questions relating to their characteristics (e.g., age, nationality and ethnicity). This was followed by questions about fairness and inclusion of DSD athletes in different contexts within elite sport, that is, sports heavily reliant on ‘physical capacity’ such as 800 m running; ‘precision sports’ such as shooting; and ‘contact sports’ such as boxing. Due to the novelty of the subject area, there were no standardised or validated questionnaires on elite athletes' opinions of DSD athletes' eligibility and fairness at the competitive level. Thus, a literature review informed the design of items and specific areas of enquiry in this mixed‐methods survey (Lundberg et al. 2018; Patton 2002; Shaw et al. 2024), which were critically evaluated by individuals not directly involved in the survey design, as in previous, similarly novel areas (Shaw et al. 2024; Braumüller et al. 2020). This evaluation was performed by three experienced academics, then by individuals known to the research team, including a gender diverse group of the general public and those competing in the female category (*n* = 11) to ensure the survey content was justifiable and respectful to achieve the best opportunity to gather participants' opinions. All relevant fairness and inclusion questions are presented in the results (Tables 1, 2, 3 or Figures 1, 2, 3). Inclusion questions exploring perspectives on DSD athletes' eligibility for elite sports were presented as a Likert‐type scale (1 = “Very Unfair” – 5 = “Very Fair”) (Sullivan and Artino 2013) or multiple choice, and an optional open text box accompanied each question to add further context (Patton 2002). Given the diversity of participant knowledge and understanding (Andreenkova and Javeline 2018), some questions were complemented with up‐to‐date information and participants had the opportunity to explain or share their thoughts in the optional open text box.

**TABLE 1:** Responses of all athletes.

Questions	*n*	All athletes (%)	
		Yes	No
Do you think sporting authorities and governing bodies could be doing more to make sports more inclusive for athletes with a DSD in terms of developing the regulations to compete?	135	82.2	17.8
Should there be a separate category of elite sports for female athletes with a DSD?			
Contact sports	117	41	59
Sports heavily reliant on physical capacity	120	40.8	59.2
Precision sports	118	30.5	69.5
Do you think the World Athletics criteria^a^ for a female elite athlete with a DSD^b^ to compete in certain athletic events are fair?	113	30.1	69.9
Do you think it is unethical to ask athletes to take nonmedically required drugs or alter prescribed medication to comply with sporting regulations?	134	67.2	32.8
Have you witnessed any negative attitudes or discrimination towards athletes with a DSD?	128	44.5	55.5

*Note: n* = number of participants. ^a^ and ^b^ were presented to respondents at the point of questioning.

^a^ Refers to the 2018 World Athletic criteria, serum testosterone below 5 nmol/L for 6 months prior to and during competition.

^b^ Athlete with a DSD refers to a relevant athlete defined in the World Athletics 2018 eligibility regulations for the female classification.

**TABLE 2:** Responses of retired Olympic (RET), current Olympic (CO) and Olympic‐recognised (OR) sport athletes.

Questions	*n*	RET (%)		*n*	CO (%)		*n*	OR (%)		
		Yes	No		Yes	No		Yes	No	
Do you think sporting authorities and governing bodies could be doing more to make sports more inclusive for athletes with a DSD in terms of developing the regulations to compete?	32	81.3	18.7	46	73.9	26.1	36	91.7	8.3	*
Should there be a separate category of elite sports for female athletes with a DSD?										
Contact sports	29	51.7	48.3	45	51.1	48.9	37	24.3	75.7	*
Sports heavily reliant on physical capacity	30	50	50	45	51.1	48.9	37	27	73	*
Precision sports	30	33.3	66.7	45	37.8	62.2	37	21.6	78.4	
Do you think the World Athletics criteria^a^ for a female elite athlete with a DSD^b^ to compete in certain athletic events are fair?	30	30	70	42	40.5	59.5	34	20.6	79.4	
Do you think it is unethical to ask athletes to take nonmedically required drugs or alter prescribed medication to comply with sporting regulations?	32	59.4	40.6	50	62	38	36	72.2	27.8	
Have you witnessed any negative attitudes or discrimination towards athletes with a DSD?	32	43.8	56.2	46	30.4	69.6	36	41.7	58.3	

*Note: n* = number of participants; differences between OR and CO are indicated by *p < 0.05. ^a^ and ^b^ were presented to respondents at the point of questioning.

^a^ Refers to the 2018 World Athletic criteria, serum testosterone below 5 nmol/L for 6 months prior to and during competition.

^b^ Athlete with a DSD refers to a relevant athlete defined in the World Athletics 2018 eligibility regulations for the female classification.

**TABLE 3:** Responses of Current Olympic (CO) sport athletes according to competitive levels (Tier 5 vs. Tier 4).

Questions	*n*	Tier 5 (%)		*n*	Tier 4 (%)	
		Yes	No		Yes	No
Do you think sporting authorities and governing bodies could be doing more to make sports more inclusive for athletes with a DSD in terms of developing the regulations to compete?	13	69.2	30.8	31	77.4	22.6
Should there be a separate category of elite sports for female athletes with a DSD?						
Contact sports	12	50	50	31	51.6	48.4
Sports heavily reliant on physical capacity	12	58.3	41.7	31	48.4	51.6
Precision sports	12	41.7	58.3	31	35.5	64.5
Do you think the World Athletics criteria^a^ for a female elite athlete with a DSD^b^ to compete in certain athletic events are fair?	12	41.7	58.3	28	35.7	64.3
Do you think it is unethical to ask athletes to take nonmedically required drugs or alter prescribed medication to comply with sporting regulations?	12	58.3	41.7	36	66.7	33.3
Have you witnessed any negative attitudes or discrimination towards athletes with a DSD?	13	38.5	61.5	31	29	71

*Note: n* = number of participants; Tier 4 = elite athletes and Tier 5 = world class athletes adapted from Mckay et al. (2022). ^a^ and ^b^ were presented to respondents at the point of questioning.

^a^ Refers to the 2018 World Athletic criteria, serum testosterone below 5 nmol/L for 6 months prior to and during competition.

^b^ Athlete with a DSD refers to a relevant athlete defined in the World Athletics 2018 eligibility regulations for the female classification.

### 2.2 Inclusion Criteria

Participants were over 18 years old, eligible for the female category before June 13, 2022, world class (Tier 5), elite (Tier 4) or national level (Tier 3) athletes (McKay et al. 2022) and gave full informed consent. As described previously (Shaw et al. 2024), participants first self‐selected as either a ‘retired elite athlete’ or ‘elite athlete’, then responses to athlete status (highest competitive achievement) were used to determine Tier 5, Tier 4 or Tier 3 competitive level, adapted from McKay et al. (2022). Athletes who were finalists in the World Games (sports that have competitions internationally but are not currently Olympic sports) were also classified as Tier 5 athletes. Athletes from sports that appeared in the Paris 2024 Olympic Games were current Olympic sport (CO) and retired Olympic sport (RET) athletes (International Olympic Committee 2024b). Athletes from sports with international federations recognition by the IOC, but that were not in the 2024 Paris Olympic Games, were Olympic‐recognised sport (OR) athletes (International Olympic Committee 2024a).

### 2.3 Statistical Analysis

Available case analysis was used and summarised descriptively using percentage values. Data are presented as mean (standard deviation), where relevant. Pearson's chi‐squared test of independence was used to compare CO versus current OR athletes, CO versus RET athletes and Tier 4 versus Tier 5 Olympic sport athletes (Sullivan and Artino 2013; Boone and Boone 2012). The maximum likelihood ratio was used when Pearson's chi‐squared assumptions were not met (Boone and Boone 2012). All tests were performed using SPSS statistics (Version 28.0.1.1, SPSS Inc., Chicago, IL) with alpha set at *p* = 0.05. Each item was considered independent to protect against type two statistical error, and alpha adjustment was not adopted (Shaw et al. 2024; Matsunaga 2007; O'Keefe 2003).

## 3 Results

### 3.1 Sample Description

A total of 147 athlete participants completed the survey. The participants consisted of 59 retired (mean age = 40 (12.1) years) and 88 current (age = 28.5 (8.7) years) athletes with a range of nationalities (United States of America 32.7%, United Kingdom 20.4%, Canada 15.7%, Finland 8.8%, Australian 2.0%, Germany 2.0%, Brazil 1.4%, the Czech Republic 1.4%, Italy 1.4%, Netherlands 1.4%, Portugal 1.4%, Russia 1.4%, South Africa 1.4%, Switzerland 1.4% and others 7.2%) and sports (Olympic *n* = 84; ice/speed skating 34.5%, curling 15.5%, athletics 11.9%, swimming 11.9%, canoeing/kayaking 10.7%, hockey 2.4%, rugby 2.4%, skiing 2.4%, others 8.3% and Olympic recognised *n* = 63; flying disc sports 87.2%, netball 4.8%, tug of war 4.8%, aerobatic pilot 1.6% and lacrosse 1.6%). The sample included 21 World Champions, 15 Olympians (two gold, one silver and three bronze medal winners) and six Paralympians (including one gold medal winner). All participants reported their sex recorded at birth as female and their gender identity as cis women (*n* = 138), nonbinary or gender neutral (*n* = 4); four individuals selected ‘other identity’ then commented that they opposed the term “cis women” or “gender identity”, and one participant selected other with no comment. No participants identified themselves as an individual with a DSD.

Most athletes agreed that sporting authorities and governing bodies could do more to make sports more inclusive for athletes with a DSD when developing regulations (82.2%; Table 1). Athletes believed DSD athletes get treated unfairly across sport in general (58.5%), whereas only 8.2% believed athletes with a DSD are treated fairly (Figure 1b). Notably, 44.5% of all athletes have witnessed negative discrimination towards DSD athletes (Table 1). Of all athletes, 42.8% believed it was fair for athletes with a DSD to compete in contact sports compared to 36.2% viewing inclusion as unfair (Figure 1a). This result was similar to sports heavily reliant on physical capacity, where 42.3% of athletes believed including DSD athletes was fair, whereas 35.5% viewed it as unfair (Figure 1a). Opinions on including athletes with a DSD in precision sports were less divided, where more respondents believed inclusion was fair than unfair (54.4% vs. 23.5%). Meanwhile, DSD athlete participation was most strongly considered unfair in the athlete participant's own sport (38.5%; Figure 1a).

Regarding the World Athletics 2018 eligibility regulations for female classification, 69.9% of respondents believed the criteria for DSD athletes required to compete in certain events were unfair (Table 1). Further, most athletes considered it unethical for DSD athletes to be obligated to use nonmedically required drugs or to alter prescribed medication to comply with sporting regulations (67.2% Table 1).

### 3.2 Current Olympic Sport (CO) Versus Olympic‐Recognised Sport (OR) Athletes

While high in both groups, a greater proportion of OR believed sporting authorities could do more to make sport more inclusive for DSD athletes (91.7%) compared to CO (73.9%; *p* = 0.039; Table 2). A higher proportion of OR thought DSD athletes were treated unfairly across all sports (72.2%) compared to CO (42.3%; *p* = 0.025; Figure 2b). Of OR athletes, 47.2% believed it very fair for DSD athletes to compete in an elite female category of their sport compared with 19.6% of CO athletes (*p* = 0.029; Figure 2a). Notably, 39.1% of CO believed it is very unfair for DSD athletes to compete in the elite female category in contact sport compared with just 13.5% of OR (*p* = 0.002; Figure 2a). Similarly, more CO than OR believed it is very unfair for DSD athletes to compete in the elite female category in sports heavily reliant on physical capacity (CO 33.3% very unfair vs. OR 13.9% very unfair, *p* = 0.003; Figure 2a).

The majority of OR (75.7%) believed there should not be a separate category for DSD athletes in contact sports compared to CO, where the opinion was more divided (48.9%; *p* = 0.013; Table 2). This result was similar to sports heavily reliant on physical capacity, where 73% of OR and 48.9% of CO athletes believed a separate category should not exist (*p* = 0.027; Table 2).

### 3.3 Current (CO) Versus Retired (RET) Olympic Sport Athletes

More RET believed DSD athletes are treated very fairly (16.7%) compared to CO (2.2%; *p* = 0.014; Figure 2b). However, 43.8% of RET athletes disclosed that they had witnessed negative attitudes or discrimination towards DSD athletes compared to 30.4% of CO (*p* = 0.228; Table 2). There was no other significant difference between CO and RET (Figure 2a, 2b and 2c; Table 2).

## 4 Discussion

This study is the first of its kind to survey the largest known sample of national, elite and world class athletes eligible to compete in the female category for their perspectives on the eligibility of DSD athletes in the female category of elite sports. Most of the 147 athletes (67.2%) believed requiring athletes to take nonmedically required drugs to comply with sporting regulations was unethical. This finding was most notable in Olympic‐recognised sports athletes (72.2%). Furthermore, 69.9% of athletes felt that the World Athletics 2018 criteria for athletes with a DSD to compete in restricted events following testosterone suppression were unfair. Given that the widely publicised World Athletics policies required DSD athletes to take such medications (World Athletics 2023), this shows a discordance between the values held by athletes and the sporting federation. In contrast, the present data show that athletes agree with the World Medical Association's stance, which states ‘Medical treatment for the sole purpose of altering the performance in sport is not permissible’ (Physician Leaders Reaffirm Opposition to IAAF Rules 2019). This finding may also contribute to our observations that most respondents believed DSD athletes were treated unfairly across elite sports.

Most athletes believed there should not be a separate category for athletes with DSDs in contact sports (59%), sports heavily reliant on physical capacity (59.2%) or precision sports (69.5%). This result provides support to those that have argued against the concept of a third category for DSD athletes (and other non‐sex binary categories), likely as the number of elite DSD athletes is too small and could also cause stigmatisation (Knox et al. 2019; Hamilton et al. 2021). Nonetheless, although most participants opposed a separate category, current Olympic sport athletes were more balanced on whether there should be a separate category in contact sports and sports heavily reliant on physical capacity (51.1% for a separate category in both; Table 2). This could possibly reflect the increased perception of DSD athlete presence in Olympic sports and the perceived competitive advantage they are proposed to possess (Tucker et al. 2024). However, very recently, the first data of known DSD athletes' in‐competition performance presented that, as a group, DSD athletes show no performance advantage over other competitors (Gollish et al. 2025), therefore showing empirically that these perceptions (Tucker et al. 2024) have been misplaced. Nevertheless, more Olympic‐recognised sport athletes view the treatment of DSD athletes as very unfair compared to retired and current Olympic sport athletes (Figure 2b). This finding could reflect the importance placed on inclusion or the current measures for DSD athlete eligibility by sports federations representing these athletes, who are likely to share some values and attitudes with their sports federation (Oliveira et al. 2023). These policies and the opinions on fairness presented herein provide insights into why negative attitudes or discrimination towards DSD athletes that have been witnessed by 44.5% of those surveyed. It must be acknowledged that no DSD athletes choose to take part in the study; however, where possible, they should be consulted on changes/developments of eligibility criteria.

While not statistically different, more retired Olympic sport athletes had witnessed negative attitudes or discrimination towards DSD athletes than current Olympic sport athletes (Table 2). This could reflect either the older age of the retired athletes simply meaning they have been in sporting environments for longer and/or a recent shift in public values towards more inclusive attitudes (Cunningham and Pickett 2018) or possibly the more subtle nature of discrimination in contemporary sports (Lashley 2022). Further, this witnessed discriminatory behaviour could partially explain the present finding that although there was a significant difference between current Olympic sport athletes (73.9%) and Olympic‐recognised sport athletes (91.7%), the majority of athletes believed sports federations could be doing more to make sports more inclusive for athletes with a DSD. This could be due to athletes acknowledging the impact that their voices may have on the decisions of sports federations and on tackling all forms of abuse and discrimination (International Olympic Committee 2021). Finally, readers should be mindful that the present sample population may not directly reflect all athlete communities that are eligible to compete in the female category, but these data represent the only currently available data of high‐level athletes' perspectives on the eligibility of DSD athletes in elite sports.

## 5 Limitations

For some respondents, there may have been limited knowledge about athletes with DSDs, the eligibility regulations and the impact that their inclusion/exclusion may have. However, a critical evaluation performed by experienced academics, a diverse group of the general public and those competing in the female category was completed before the survey's release. Further, DSD specific statements were included in the pre‐participation documents and questions were accompanied by “notes” consisting of additional information to aid understanding as much as possible. When interpreting the presented data, readers should be mindful that the sample population may not be directly reflective of all athlete communities that are eligible to compete in the female category. The term difference in sex development (DSD) was used within this study to remain consistent with scientific and medical literature and in the hope of being acceptable to the individuals the term represents (Bennecke et al. 2021). It must be acknowledged that no known DSD athletes took part in the study, and thus the data herein do not describe the views of athletes with DSDs. Finally, the survey data were collected between 2021 and 2022, and therefore the presented data were representative of opinions at that time and may or may not be reflective of views at the point of publication.

## 6 Conclusion

The present study is the first to report on the largest known sample of opinions from national, elite and world class athletes regarding the eligibility of DSD athletes in elite sports. Overall, athletes did not favour a separate category for DSD athletes, more considered their inclusion in the female catagory as fair and only a very small proportion of respondents believed DSD athletes are treated fairly. A substantial proportion of athletes had witnessed negative attitudes or discrimination towards DSD athletes, and this observation was most common in retired Olympic sport athletes. The athletes' voice has been described as ‘a truly qualified knowledge expert’ (Weissensteiner 2015) and, in combination with other scientific evidence, should be utilised to create appropriate policies that are evidenced‐based and align with the collective values of the athletes (International Olympic Committee 2021). Sports federations must ensure that policies reflect the athlete voice and understand that views differ between athlete groups and sports. Future research should explore why different groups of athletes have varied opinions on the eligibility of DSD athletes such as race/geographic ancestry, economic incentives, intrinsic beliefs on sex–gender etc., and explore the views of athletes with DSDs themselves regarding the eligibility regulations that affect them most directly.

## Ethics Statement

Ethical approval was granted by the Faculty of Science and Engineering Research Ethics and Governance Committee, Swansea University (SU‐Ethics‐Staff‐210622/486).

## Conflicts of Interest

AS, GS, MC, LC and SH have no competing interests to declare.

AH identifies as LGBTQI+ and volunteers at an LGBTQI+ library in London. AW received travel/accommodation/honorarium for speaking at three relevant events during 2022–23 (Sport Resolutions Annual Conference; Global Observatory for Gender Equality & Sport First International Conference on Inclusive Gender Equality: Open Fields, Open Questions; IOC Framework on Fairness, Inclusion and Nondiscrimination Workshop. AW has served as an independent expert witness in relevant cases at the Court of Arbitration for Sport in 2019 and 2024). NF has consulted for the Canadian Centre for Ethics in Sport (CCES) and Athlete Ally, where he critiqued research impacting athletes with a DSD.

## Data Availability

All data are available on reasonable request.
